# Prediction of pituitary adenoma surgical consistency: radiomic data mining and machine learning on T2-weighted MRI

**DOI:** 10.1007/s00234-020-02502-z

**Published:** 2020-07-23

**Authors:** Renato Cuocolo, Lorenzo Ugga, Domenico Solari, Sergio Corvino, Alessandra D’Amico, Daniela Russo, Paolo Cappabianca, Luigi Maria Cavallo, Andrea Elefante

**Affiliations:** 1 Department of Advanced Biomedical Sciences, University of Naples “Federico II”, Via Pansini, 5, 80131 Naples, Italy; 2 Department of Neurosciences, Reproductive and Odontostomatological Sciences, Division of Neurosurgery, University of Naples “Federico II”, Naples, Italy

**Keywords:** Machine learning, Radiomics, Magnetic resonance imaging, Pituitary adenoma, Consistency

## Abstract

**Purpose:**

Pituitary macroadenoma consistency can influence the ease of lesion removal during surgery, especially when using a transsphenoidal approach. Unfortunately, it is not assessable on standard qualitative MRI. Radiomic texture analysis could help in extracting mineable quantitative tissue characteristics. We aimed to assess the accuracy of texture analysis combined with machine learning in the preoperative evaluation of pituitary macroadenoma consistency in patients undergoing endoscopic endonasal surgery.

**Methods:**

Data of 89 patients (68 soft and 21 fibrous macroadenomas) who underwent MRI and transsphenoidal surgery at our institution were retrospectively reviewed. After manual segmentation, radiomic texture features were extracted from original and filtered MR images. Feature stability analysis and a multistep feature selection were performed. After oversampling to balance the classes, 80% of the data was used for hyperparameter tuning via stratified 5-fold cross-validation, while a 20% hold-out set was employed for its final testing, using an Extra Trees ensemble meta-algorithm. The reference standard was based on surgical findings.

**Results:**

A total of 1118 texture features were extracted, of which 741 were stable. After removal of low variance (*n* = 4) and highly intercorrelated (*n* = 625) parameters, recursive feature elimination identified a subset of 14 features. After hyperparameter tuning, the Extra Trees classifier obtained an accuracy of 93%, sensitivity of 100%, and specificity of 87%. The area under the receiver operating characteristic and precision-recall curves was 0.99.

**Conclusion:**

Preoperative T2-weighted MRI texture analysis and machine learning could predict pituitary macroadenoma consistency.

**Electronic supplementary material:**

The online version of this article (10.1007/s00234-020-02502-z) contains supplementary material, which is available to authorized users.

## Introduction

Pituitary adenomas are frequent tumors of the pituitary gland. Although most pituitary macroadenomas have a soft consistency, some are rather fibrous and therefore more challenging to remove by transsphenoidal adenomectomy. Indeed, tumor consistency has been reported as one of the principal determinants of transsphenoidal surgery success rate [1]. For this reason, the ability to preoperatively assess adenoma consistency could improve surgical planning and reduce complication rate and risk of residual tumor presence [2].

Radiomics, consisting of conversion of images into mineable data and subsequent analysis for decision support, has been gaining attention in recent years [3]. In particular, texture analysis is a post-processing technique allowing for quantitative description of pixel gray-level heterogeneity. More recently, texture analysis-derived features have been used in association with data mining and machine learning algorithms, aiding in the interpretation of a large amount of information produced. Machine learning (ML) is the branch of artificial intelligence including algorithms capable of modeling themselves and improving in accuracy by analyzing datasets, without prior explicit programming [4]. It leads to the creation of predictive models that are able, among other tasks, to solve classification problems. The usefulness of the radiomic approach is being assessed in different fields of radiology [5–9].

Our aim was to assess the accuracy of a ML model trained on radiomic data mined from MRI exams to predict pituitary macroadenoma surgical consistency prior to an endoscopic endonasal procedure.

## Methods and materials

### Patient population

This retrospective study was conducted in accordance with the 1964 Helsinki Declaration and its later amendments. The local Institutional Review Board gave its approval and waived the need for informed consent. We reviewed all patients referred to our institution for endoscopic endonasal pituitary adenoma removal (January 2013–December 2017). Those with history of previous treatment for pituitary adenoma (radiation or medical therapy) at the time of MRI, lesions smaller than 10 mm, extensively necrotic or hemorrhagic areas, or significant artifacts on the images used for the analysis were excluded.

### Consistency assessment

All patients were operated on by two neurosurgeons with over 10 years of experience in a third level referral center in the field of pituitary surgery [10]. Tumor consistency, classified as soft or fibrous, was assessed in blinded double-check by the two surgeons according to the lesions’ inner surgical features. In detail, adenomas easily removable with conventional maneuvers of curettage and suction were defined as soft. More resistant ones, difficult to remove and thus requiring more complex maneuvers such as extracapsular dissection, were classified as fibrous [11–14]. Surgical features of soft and fibrous pituitary macroadenomas are depicted in online Video 1 and 2, respectively.

ESM 2(MOV 17822 kb)ESM 3(MOV 25323 kb)

### Image acquisition

All patients underwent MRI exams either on a 1.5 (Gyroscan Intera, Philips, Eindhoven, the Netherlands) or 3 T MR scanner (Magnetom Trio, Siemens Medical Solutions, Erlangen, Germany). The imaging protocol always included a coronal T2-weighted (T2-w) Turbo Spin Echo sequence whose detailed parameters are reported in supplementary Table 1.

### Handcrafted radiomics

**Fig. 1:**
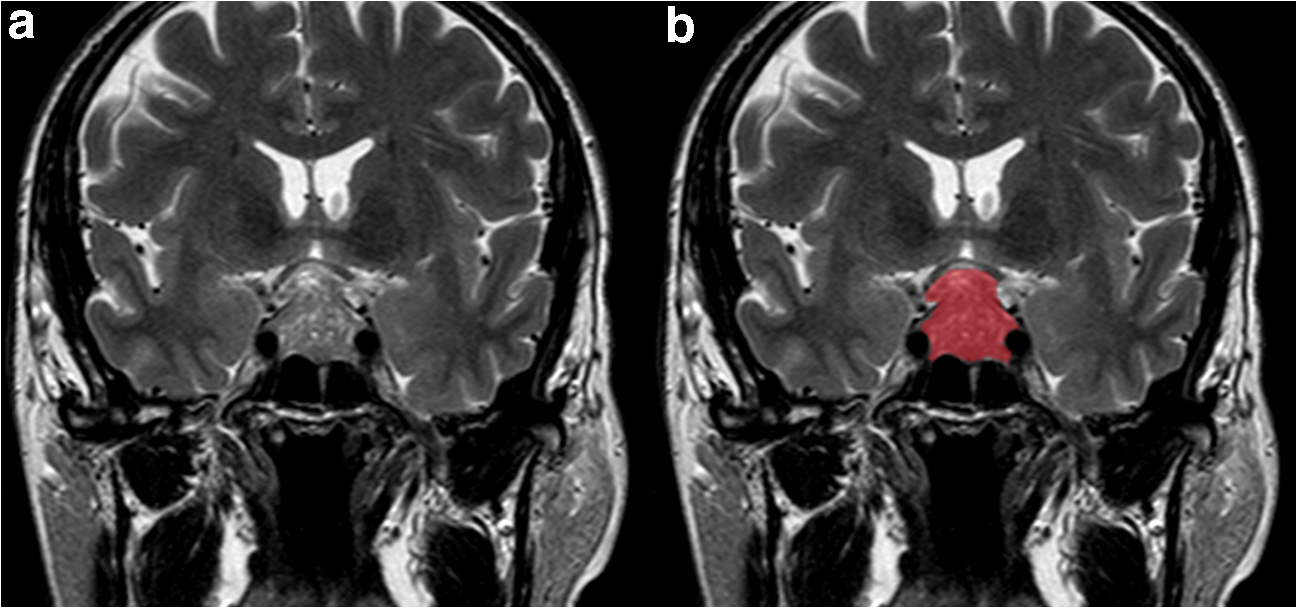
Pituitary macroadenoma segmentation example on coronal T2-weighted (**a**), showing hand-drawn ROI placement (**b**)

Adenomas are manually annotated by a neuroradiologist (8 years of experience) by placing a 2D polygonal region of interest (ROI) on the coronal slice of maximum lesion extension on a freely available segmentation software (ITKSnap v3.8.0) (Fig. 1). Two other readers (both > 5 years’ experience) also performed lesion segmentation on all patients, blinded to the first neuroradiologist’s ROI placement, to perform radiomic feature stability testing.

A freely available, well-established, and open-source Python software platform was used for image pre-processing and radiomic parameter extraction (Pyradiomics, v2.2.0). First of all, images and ROIs were resampled to a 2 × 2 × 2 mm isotropic voxel, as required for further pre-processing (i.e., correct use of image filters). All voxel intensity values were also normalized by subtracting the mean intensity and dividing by the standard deviation and discretized by using a fixed bin width (=3). Filtered images were also employed for feature extraction in addition to the pre-processed original T2-w ones. In particular, a Laplacian of Gaussian filter, with sigma values ranging from 2.0 (most fine texture) to 3.5 (most coarse) in 0.5 increments, and all available combinations of wavelet decomposition high- and low-pass filtering in the *x*, *y*, and *z* dimensions were applied. While 2D ROIs were drawn, we still chose to employ a three-dimensional wavelet decomposition as after resampling the software will detect an ROI *z*-axis value > 1. This is not an issue for the analysis as we excluded shape features, and the following feature selection steps will remove all redundant parameters that could have been extracted from similar wavelet decomposition-derived images.

### Data mining and machine learning

Initial assessment and processing of the extracted data were performed on Python in particular using the numpy, pandas, and scikit-learn packages. First of all, the intraclass correlation coefficient (ICC) was calculated for each parameter as extracted using ROIs from the three readers. A two-way, absolute agreement and single rater ICC was employed, and only features with values ≥ 0.75 were considered stable. Non-informative, low variance (variance ≤ 0.1) features were also excluded from the dataset. Then, a pairwise correlation matrix was calculated for these in order to remove all features with an intercorrelation ≥ 0.8. As we expect an unbalanced dataset due to the relative rarity of fibrous adenomas compared with soft ones, the Synthetic Minority Oversampling Technique (SMOTE) was employed [15]. Then, 80% of the data was used for hyperparameter tuning via stratified 5-fold cross-validation, while a 20% hold-out set was employed for its testing on unseen data. In detail, the following steps were exclusively performed on the first set. A normalization scaler was calculated to remove biases due to feature scale and was later applied to the hold-out test set. Finally, recursive feature elimination (RFE), employing a logistic regression algorithm and stratified 5-fold cross-validation, was used to select the better performing feature subset.

The resulting data was used to train an ensemble learning meta-algorithm, the Extra Trees Classifier (ET). These often demonstrate good performance on radiomic medical image data [16]. Its performance for consistency prediction was finally assessed on the test set.

Accuracy metrics were obtained using the scikit-learn package and further analyzed on the R software (R for Unix/Linux, version 3.4.4, the R Foundation for Statistical Computing, 2014). In particular, DeLong’s test (pROC package) was used to obtain 95% confidence intervals (95%CI) of the area under the receiver operating characteristic curve (AUC) and the confusion matrix function (caret package) those of the classifier’s accuracy and compare its performance to the no information rate.

**Fig. 2:**
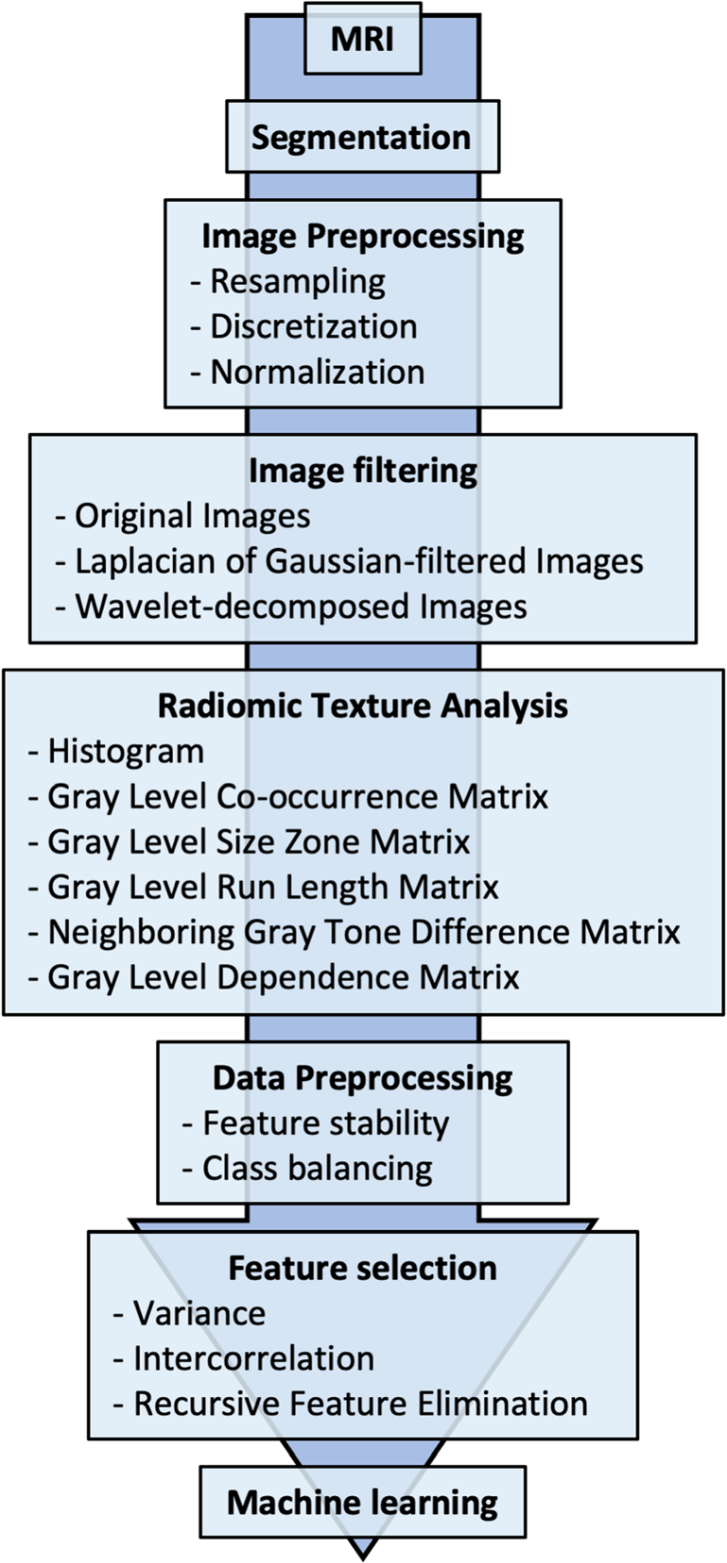
Radiomic workflow pipeline

The described radiomics workflow pipeline is illustrated in Fig. 2.

## Results

According to selection’s criteria, 89 patients were included in this study; 51 were males and 38 females, with mean age 52.17 ± 14.64 years (range 16–80). Average lesion size was 25 ± 8 mm (range 8–46 mm). The pituitary lesions were classified as soft in 68 patients and fibrous in the remaining 21. In detail, 19 soft (8 ACTH, 7 GH, 4 PRL) and 6 fibrous (2 ACTH, 2 PRL, 1 GH, 1 TSH) were functioning (25/89, 28% in total). In none of the cases, there was discordance among the neurosurgeons in lesion classification. Patient population clinical data are presented in Table 1.

**Table 1:** Patient population clinical data

	Tumor consistency		
	Total (*n* = 89)	Soft (*n* = 68)	Fibrous (*n* = 21)
Age (mean) (year)	52.2 ± 14.6	53.2 ± 15.5	54.6 ± 14.6
Sex			
Males (*n*) (%)	51 (57%)	39 (57%)	12 (57%)
Females (*n*) (%)	38 (43%)	29 (43%)	9 (43%)
Tumor type			
Functioning (*n*) (%)	25 (28%)	19 (28%)	6 (21%)
Non-functioning (*n*) (%)	64 (72%)	49 (72%)	15 (78%)

**Fig. 3:**
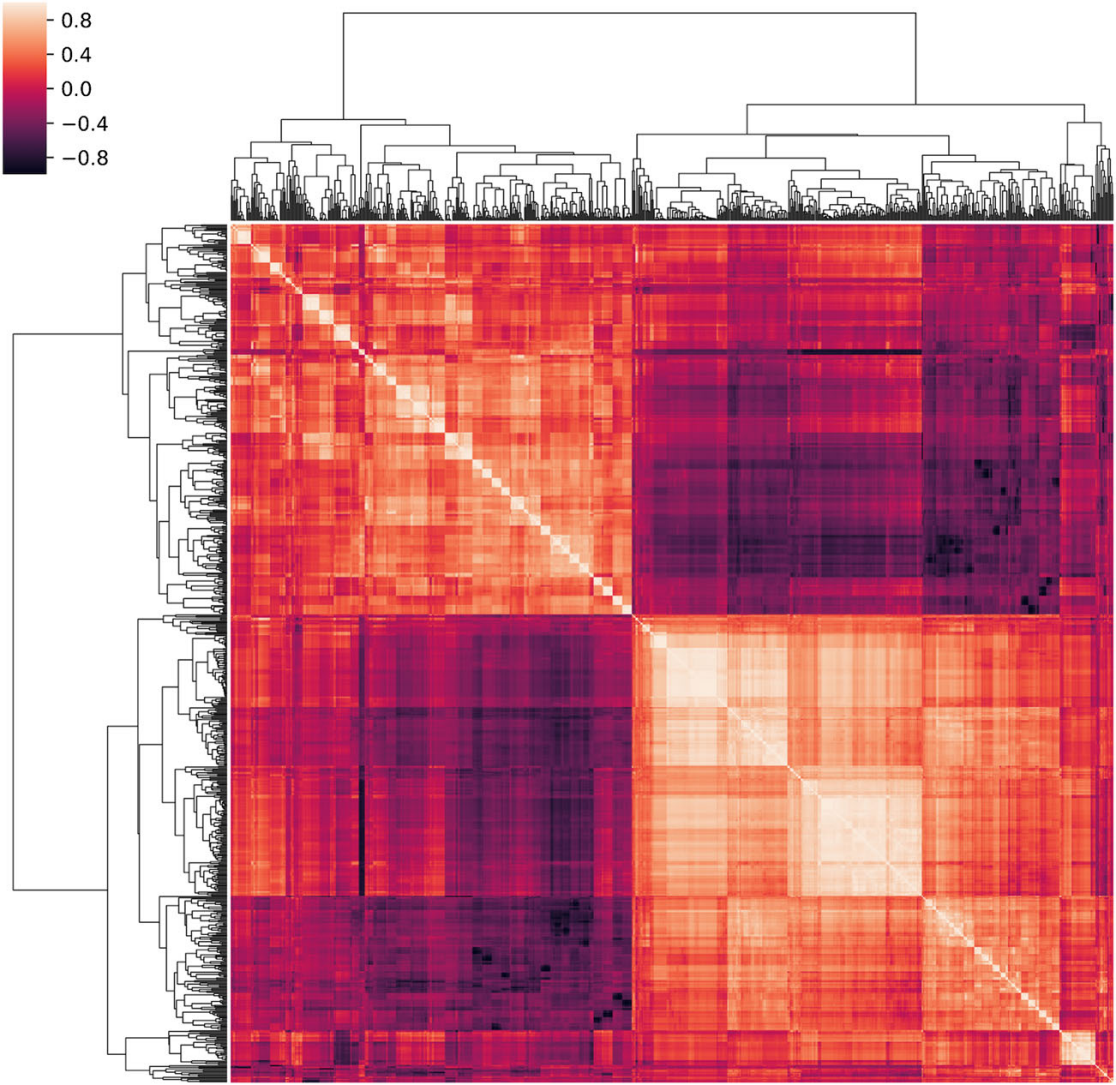
Hierarchically clustered heatmap of the feature correlation matrix. Features with an intercorrelation above the selected threshold (≥ 0.8) were removed from the dataset

**Fig. 4:**
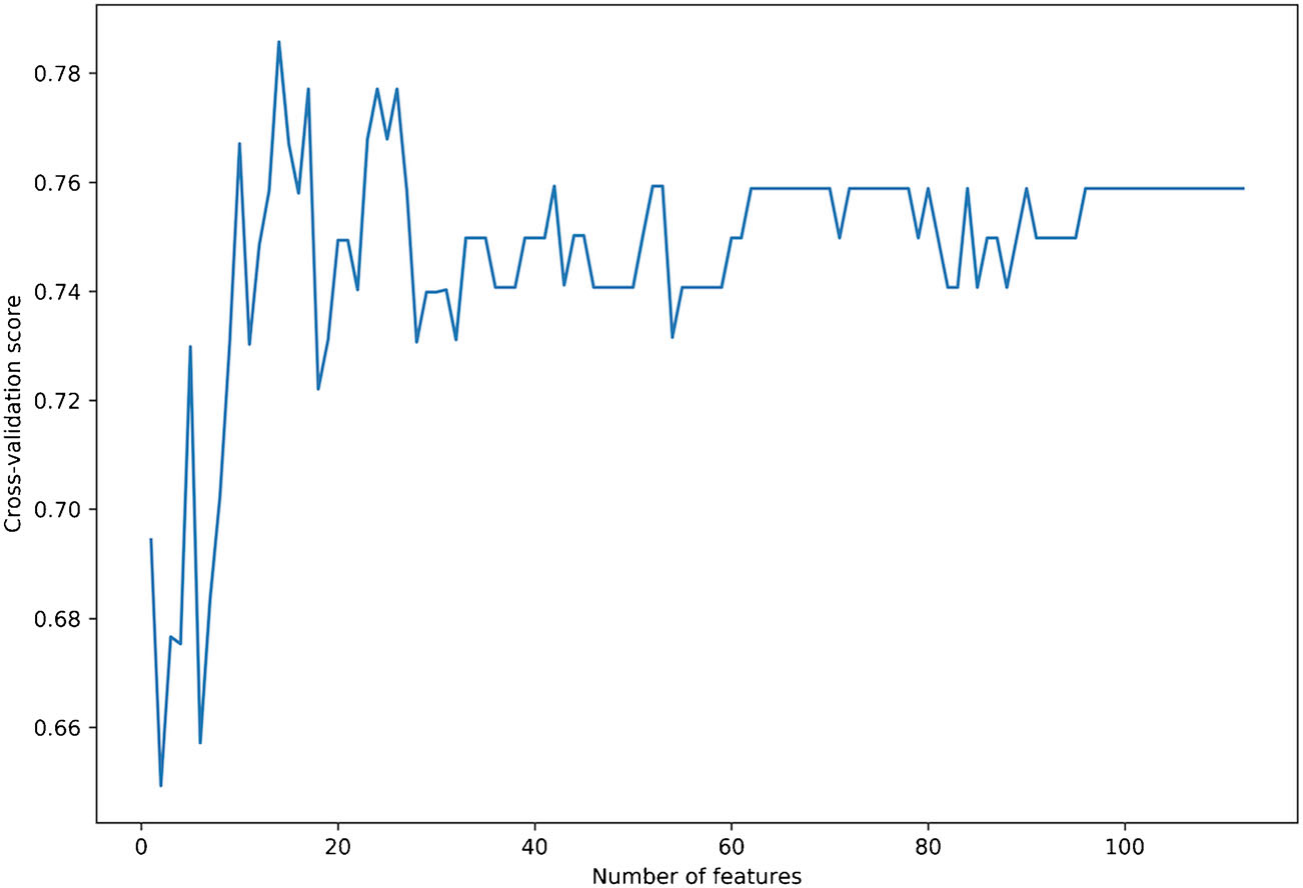
Plot of the feature selection process by recursive feature elimination. The *x*-axis contains the total number of features, from which one is removed at each iteration. The *y*-axis contains the average cross-validation score for each feature total

A total of 1118 texture features were extracted, including first- and higher-order texture features from the original and filtered images. The correlation cluster map of the extracted features is shown in Fig. 3. Their detailed description is available in the online Pyradiomics documentation (https://pyradiomics.readthedocs.io/en/latest/features.html). After feature stability analysis, 741 were retained for the subsequent steps. Of these, 4 had low variance, while 625 were highly intercorrelated. RFE then identified a 14-feature subset as most accurate (Fig. 4; feature list is available in supplementary material).

**Fig. 5:**
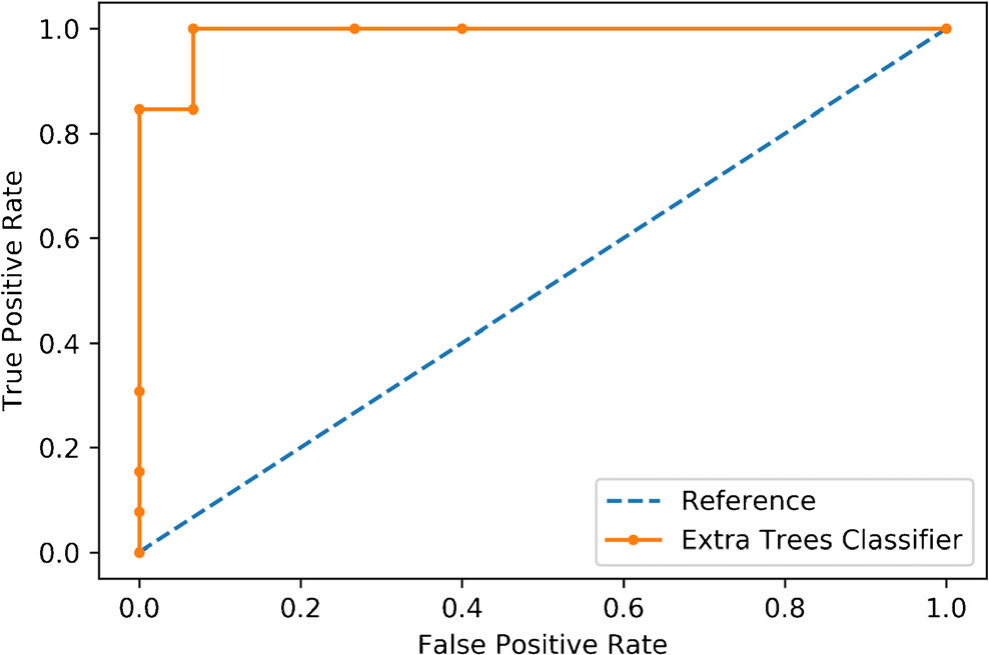
Receiver operating characteristics curve of the Extra Trees classifier accuracy

The ET model obtained an overall accuracy, in terms of correctly classified lesions, of 86% (± 10%) in the training set cross-validation. The classifier tuned parameters are reported in the supplementary materials. In the test set, the accuracy was of 93% (95%CI = 77–99%), sensitivity of 100%, and specificity of 87%. The AUC is of 0.99 (95%CI = 0.97–1.00) (Fig. 5), equal to the area under the precision-recall curve (0.99), often used in binary ML classifications (fig AUC). The classifier was significantly better (*p* = 8e^−6^) than the no information rate. The confusion matrix and detailed accuracy metrics are shown in Tables 2 and 3.

**Table 2:** Confusion matrix for the test group

		Predicted class	
		Soft	Fibrous
Actual class	Soft	13	2
	Fibrous	0	13

**Table 3:** Extra Trees classifier accuracy metrics

Class	Recall	Precision	*F*-score	AUC	AUPRC
Soft	0.87	1.00	0.93	0.99	0.99
Fibrous	1.00	0.87	0.93	0.99	0.99
WAvg	0.94	0.93	0.93	0.99	0.99

*WAvg* weighted average, *AUC* area under the receiver operating characteristic curve; *AUPRC* area under the precision-recall curve

## Discussion

Preoperative assessment of pituitary macroadenoma consistency is useful for planning surgical approach and reducing residuals and recurrence’s rate. For this reason, several studies have investigated the correlation between preoperative MRI features and tumor hardness. In particular, there are conflicting studies on the value of the relative signal on T2-weighted MRI and the macroadenoma consistency, with some works demonstrating a positive correlation between low signal and hardness [17–20] and other concluding that relative signal intensity values do not correlate [21–24]. Indeed besides collagen amount, which mainly correlates with the hardness, other factors such as intratumoral hematoma, amyloid, iron, calcification, or protein-rich fluid may affect the T2 signal intensity [25].

Diffusion-weighted imaging ability to predict tumor consistency also showed divergent results, both indicating a significant correlation [1, 26] and not [21, 27]. Furthermore, the lower spatial resolution and the presence of susceptibility artefacts in the sellar region related to bone and sinus pneumatization limit the use of this technique. Finally, in two studies by Romano et al. and Yamamoto et al., contrast-enhanced MRI showed a strong correlation for tumor consistency [2, 28]. Perfusion imaging parameters have also been investigated as possible biomarkers of pituitary macroadenoma consistency, but no added value was found compared with precontrast T1-weighted images [29]. A more interesting advanced technique in this setting is represented by MR elastography. Pituitary adenoma stiffness was found to correlate with their consistency and, if it became widely available, could offer additional data to mine with a radiomic approach [30, 31].

Regarding texture analysis, there are only three studies exploring this issue, to the best of our knowledge. In the first, Rui et al. explored the value of MRI texture analysis in assessing pituitary macroadenoma consistency, obtaining good accuracy values [32]. However, this study was conducted using contrast-enhanced 3D-SPACE images and without a ML approach. Fan and colleagues explored this issue in acromegalic patients using ML for radiomic feature selection prior to building a nomogram obtaining an AUC of 0.81 [33]. Zeynalova et al. also performed an analysis on ML preoperative evaluation of pituitary macroadenoma consistency [34]. Their study presented some similarities with our own. They also used Pyradiomics for feature extraction from bidimensional ROIs, although they utilized different sigma settings (2, 4, and 6 mm) for the LoG filter and obtained a total of 162 parameters. The lower number of features is probably due to their exclusive focus on first-order histogram-derived ones. These are more reproducible but convey less information on tissue texture compared with higher-order parameters. As their in-plane resolution was higher (0.5 × 0.8 mm), they were able to use a 1 × 1 mm resampling size compared with our 2 × 2 mm. It is interesting to note the use of a very narrow bin width value of 0.06, as the number of bins should not exceed 128, following the developer recommendations. They also performed a feature robustness assessment with our same ICC threshold, while their intercorrelation threshold was lower (0.7 vs 0.8). After data dimensionality reduction, they identified 6 informative features using the Weka data mining platform and a wrapper-based selector. In our study, the entire analysis was conducted using the scikit-learn Python package. Some other major differences are represented by the use of cross-validation, without further assessment on a separate test set. Their reported accuracy is 72.5%, with an AUC of 0.71. Therefore, our algorithm presents a clearly superior performance. This could be in part explained by their use of a multilayer perceptron neural network, which may not be the best suited algorithm for a small dataset obtained from 55 patients. Finally, Zenyalova and colleagues also used collagen amount within the tumor on histopathological examination for their reference standard. As consistency information is mainly useful for surgical strategy planning, we believe that intraoperative consistency assessment represents a more practical and useful reference standard as the final recipient of the information should be a neurosurgeon. As the two neurosurgeons involved in our study never had disagreements, we also found this assessment to be reproducible.

By analyzing our confusion matrix, it can be seen that the mistakes made by the classifier were 2 cases of soft lesions identified as fibrous. Given the clinical setting of our investigation, this kind of error is somewhat more acceptable than a false negative, as it would be more auspicable to sometimes overestimate the difficulty of a surgery rather than the opposite.

In our study, we chose to employ an ET ML algorithm. This belongs to the decision tree ensemble methods, in particular constituted by a large number of highly randomized decision trees which are fitted on data subsamples. Each of these outputs a prediction, and a majority vote determines the final outcome. Ensemble learning is based on the assumption that a decision by committee made by a large number of weak classifiers will perform better than a single algorithm. A sufficient diversification of the random trees included in the ET is guaranteed by random sampling, with replacement, of patients from the training dataset (bootstrap aggregation or bagging) and of their available features (*n* = 3 in our case). This in turn ensures low correlation of each tree, improving the ET’s overall performance [16]. As the dataset lesion classes were imbalanced, SMOTE was employed. This is a known solution to address this issue and has demonstrated its value in the setting of medical imaging radiomic ML analysis [15, 35–37].

We have chosen a handcrafted radiomics approach rather than a fully automated deep learning one as this gave us better control on the initial data analysis and following ML model construction. Both approaches have been object of discussion in current literature as they possess peculiar merits and limitations. It is our belief that a handcrafted analysis is more appropriate for relatively smaller datasets as it allows greater involvement of radiologists and better understanding of the whole pipeline. Only when extremely large datasets will become available in medical imaging, as in other fields, the less time-consuming completely neural network-based approach will be a practical necessity. Until then, the value of greater involvement of the radiologist and finer quality control of patient or lesion data outweigh the larger amount of time needed to extract medical imaging radiomics. Furthermore, medicine and especially treatments are evolving in the direction of precision, patient-tailored therapies. Contrary to the current desire in radiology to aggregate as many patients as possible to train ML algorithms, this determines a need to work with ever smaller patient subgroups within each pathological entity. Therefore, a future with space for both deep learning software to apply on large populations and engineered approaches for more specific tasks can be envisioned.

Our study has some limitations which have to be acknowledged. As is often the case for ML, future studies on larger populations are necessary to confirm and possibly expand our results. The need for oversampling given the unbalanced nature of the classes further highlights this necessity but was expected given epidemiological data. Only T2-weighted images were used, without investigating the added value of other sequences. However, obtaining valuable data without contrast agent administration and streamlining the pipeline to incorporate a single MRI sequence could also represent an added value. Furthermore, considering previous works, T2-weighted MRI alone proved effective to provide data concerning proliferative index [38], secretory activity [39], and response prediction to somatostatin analogues in patients with acromegaly and GH secreting pituitary macroadenoma [40, 41].

## Conclusion

The ML model trained on radiomic data extracted from T2-weighted MRI demonstrated a high accuracy in the classification of soft and fibrous pituitary macroadenomas. Therefore, this tool could prove valuable in the pre-surgical planning of these patients if further developed and validated on larger datasets.

## Electronic supplementary material

ESM 1 (DOCX 15 kb)

## Abbreviations

ML: Machine learning
ROI: Region of interest
ICC: Intraclass correlation coefficient
SMOTE: Synthetic Minority Oversampling Technique
RFE: Recursive feature elimination
ET: Extra Trees Classifier
AUC: Area under the receiver operating characteristic curve
