# Supplementary material for: Prediction of pituitary adenoma surgical consistency: radiomic data mining and machine learning on T2-weighted MRI
Source: Neuroradiology. 2020 Jul 23;62(12):1649–56. doi: 10.1007/s00234-020-02502-z (PMC7666676; doi:10.1007/s00234-020-02502-z)
Supplement: Supplementary file 3 — (DOCX 15 kb) [file 234_2020_2502_MOESM1_ESM.docx]

**Pituitary adenoma surgical consistency prediction on T2-weighted MRI: a radiomics machine learning analysis**

**Supplementary material**

**On-line Video 1**

Surgical procedure for the removal of a soft pituitary macroadenoma.

**On-line Video 2**

Surgical procedure for the removal of a fibrous pituitary macroadenoma.

**Supplementary table 1**

Coronal T2-weighted Turbo Spin Echo sequence parameters for 1.5 and 3 Tesla scanners.

|  | **TR** | **TE** | **FOV** | **matrix** | **thk** | **ETL** | **Slice gap** | **Acquisition time** |
| --- | --- | --- | --- | --- | --- | --- | --- | --- |
| **1.5 Tesla** | 2600 ms | 89 ms | 180x180 mm | 288x288 | 3 mm | 17 | no gap | 2 min 17 s |
| **3 Tesla** | 3000 ms | 98 ms | 200x200 mm | 384x384 | 3 mm | 18 | no gap | 3 min 22 s |

TR: Repetition Time; TE: Echo Time; FOV: Field of View; thk: slice thickness; ETL: echo train length.

**Recursive feature elimination (RFE)-identified feature subset**

1. Original firstorder Energy
2. Original firstorder Kurtosis
3. Original firstorder Skewness
4. Log-sigma-3-0-mm-3D firstorder Skewness
5. Log-sigma-3-0-mm-3D glcm Imc1
6. Log-sigma-3-0-mm-3D glszm SizeZoneNonUniformityNormalized
7. Log-sigma-3-5-mm-3D firstorder Minimum
8. Wavelet-LHL firstorder InterquartileRange
9. Wavelet-LHL gldm DependenceVariance
10. Wavelet-LHH firstorder 10Percentile
11. Wavelet-LHH firstorder Maximum
12. Wavelet-HLH firstorder Maximum
13. Wavelet-HLH firstorder Mean
14. Wavelet-HLH firstorder Minimum

**Algorithm**

Extra Trees Classifier output code after fitting on the training population in scikit-learn.

ExtraTreesClassifier(bootstrap=True, ccp_alpha=0.0, class_weight=None, criterion='entropy', max_depth=None, max_features=3, max_leaf_nodes=None, max_samples=None, min_impurity_decrease=0.0, min_impurity_split=None, min_samples_leaf=1, min_samples_split=2, min_weight_fraction_leaf=0.0, n_estimators=100, n_jobs=None, oob_score=False, random_state=None, verbose=0, warm_start=False)
